# Supplementary material for: Splice-Junction-Based Mapping of Alternative Isoforms in the Human Proteome
Source: Cell Rep. Author manuscript; Available in PMC 2020 Jan 15. (PMC6961840; doi:10.1016/j.celrep.2019.11.026)
Supplement: 2 [file NIHMS1546469-supplement-2.zip › DF1/PXD009737/TestisTrypsin/TestisTrypsin_43_KIAA1210_SLTATQVEPK.pdf]

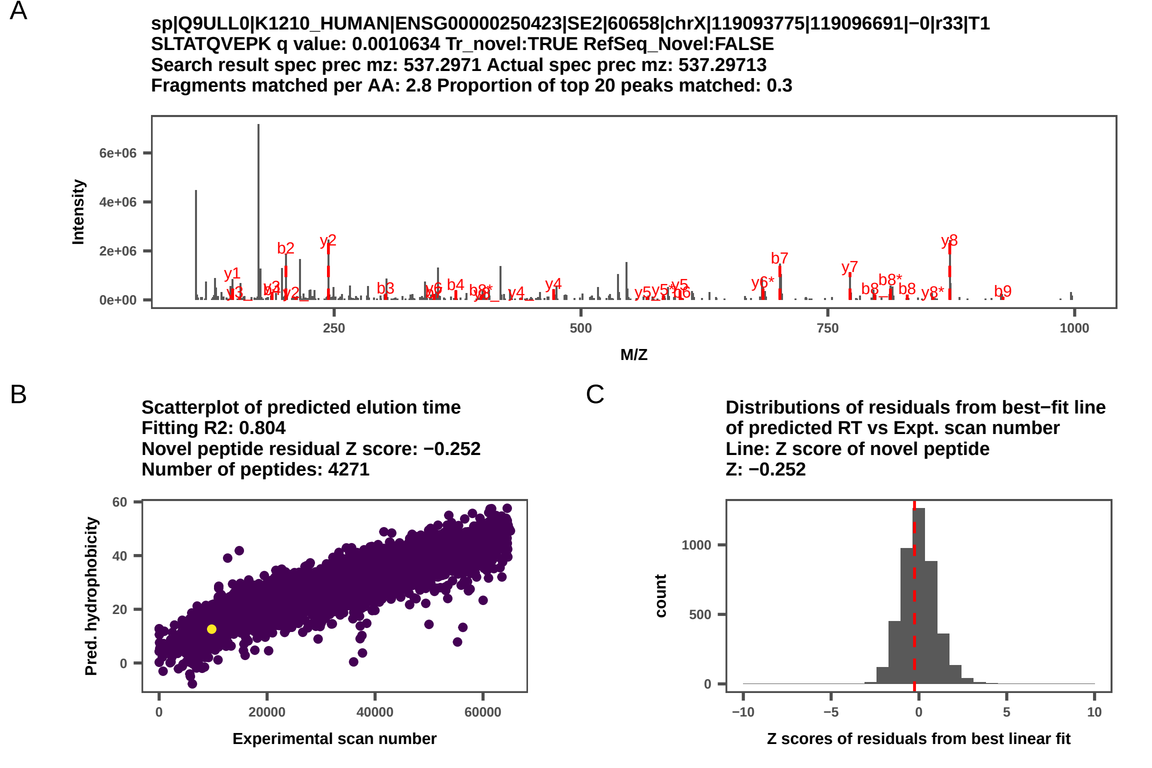
A
sp|Q9ULL0|K1210_HUMAN|ENSG00000250423|SE2|60658|chrX|119093775|119096691|−0|r33|T1
SLTATQVEPK q value: 0.0010634 Tr_novel:TRUE RefSeq_Novel:FALSE
Search result spec prec mz: 537.2971 Actual spec prec mz: 537.29713
Fragments matched per AA: 2.8 Proportion of top 20 peaks matched: 0.3
Intensity
6e+06
4e+06
2e+06
0e+00
M/Z
250
500
750
1000
y1
b2
y2
b3
b4
y4
b7
y6*
y7
b8*
b8
y8*
y8
b9
B
Scatterplot of predicted elution time
Fitting R2: 0.804
Novel peptide residual Z score: −0.252
Number of peptides: 4271
Pred. hydrophobicity
60
40
20
0
Experimental scan number
0
20000
40000
60000
C
Distributions of residuals from best−fit line
of predicted RT vs Expt. scan number
Line: Z score of novel peptide
Z: −0.252
count
1000
500
0
Z scores of residuals from best linear fit
−10
−5
0
5
10
